# Health economics and vaccine financing in the eastern Mediterranean region: A needs assessment

**DOI:** 10.1016/j.vaccine.2025.127780

**Published:** 2025-10-24

**Authors:** Palwasha Anwari, Gerald Sume, Wedyan Meshreky, Nathalie Vande Maele, So Yoon Sim, Karene Hoi Ting Yeung, Diana Kizza, Philipp Lambach, Maarten Paul Maria Jansen, Raymond Hutubessy, Quamrul Hasan

**Affiliations:** a Immunization Vaccine Preventable Disease and Polio Transition Unit, Department of Communicable Diseases, WHO Regional Office of the Eastern Mediterranean Region, Cairo, Egypt; b Department of Immunization, Vaccines and Biologicals, World Health Organization, Geneva, Switzerland; c United Nations Children Fund (UNICF) Regional Office for North Africa and Eastern Mediterranean

**Keywords:** Health economics, Immunization, Vaccine financing, Needs assessment, Eastern Mediterranean region, National Immunization Technical Advisory Groups (NITAGs)

## Abstract

**Introduction:**

The World Health Organization Eastern Mediterranean Region (EMR) faces a high burden of vaccine-preventable diseases requiring efficient use of limited resources. A regional needs assessment was conducted to evaluate the current application of health economics and vaccine financing in national immunization programmes and policy formulation, identify capacity gaps, and inform tailored technical support.

**Methods:**

A structured online survey was administered between January 28 and February 18, 2025. It targeted expended programme on immunization (EPI) managers and National Immunization Technical Advisory Group (NITAG) chairs across all 22 EMR countries. The questionnaire explored five thematic areas: current capacity in health economics and vaccine financing, training and capacity-building needs, demand for technical support, data availability and use in decision-making, and strategic planning for vaccine financing among immunization stakeholders at Ministry of Health and in NITAGs. The survey used multiple-choice, Likert-scale, and open-ended questions. Results were analyzed using descriptive statistics and disaggregated by income level.

**Results:**

The response rate was 73 % (16/22 countries). Only three countries (19 %) reported full integration of health economics within their immunization programmes, while 56 % (*n* = 9) reported minimal or no integration. Three countries had a dedicated health economics focal person for immunization. In two high-income countries immunization programmes demonstrated stronger capacity in health economics, whereas the rest of countries demonstrated low to moderate levels. Ten countries (63 %) rated their capacity in financial forecasting and planning for immunization as moderate or high, but eleven (69 %) rated their capacity in cost-effectiveness analysis and vaccine financial sustainability as low to none. Access to data varied, with 25 % of countries finding it easily accessible.

**Conclusion:**

The findings highlight the need to strengthen the integration of health economics and vaccine financing into immunization programmes and policy decision-making across the EMR, irrespective of income level. Tailored capacity-building, technical support, and cross-sectoral collaboration, particularly with academia, are essential.

## 1 Introduction

Globally, immunization programmes have proven both lifesaving and cost-saving. The expanded programme on immunization (EPI) has saved an estimated 154 million lives in the past 50 years, with measles vaccination alone accounting for over 93 million lives saved [1]. Childhood immunization yields a return of approximately $26 return for every $1 invested, reflecting both healthcare savings and productivity gains [2]. The Immunization Agenda 2030 (IA2030) defines what needs to happen to achieve the IA2030 vision of a world where everyone, everywhere, at every age fully benefits from vaccines for good health and well-being [3]. It emphasizes country ownership, data-driven decision-making, and context-appropriate strategies as key pillars to achieving equitable and sustainable immunization outcomes [3].

In the World Health Organization (WHO) Eastern Mediterranean Region (EMR), which comprises 22 countries and territories across the Middle East, North Africa, and parts of South Asia (Annex 1), these global goals are contextualized through a regional strategic framework [4]. The EMR is marked by wide disparities in income levels, health system capacities, and disease burdens, from high-income Gulf states to conflict-affected and fragile settings [5]. In the EMR, while the EPI has led to significant gains in immunization coverage and the introduction of new vaccines, progress has stalled since 2019. The region continues to face a high burden of vaccine-preventable diseases (VPDs), e.g., accounting for nearly 13 % of global measles cases in 2023 [6]. The economic toll is substantial, with treatment costs for measles complications ranging from US$150–500 per case [7] and vaccine-preventable cancer-related deaths resulting in US$1.7 billion in lost productivity across the Middle East and Northern Africa in 2024 [8]. Low coverage of vaccination has driven recurrent outbreaks, leading to avoidable treatment costs, mortality, and productivity losses [9].

A National Immunization Strategy (NIS) is a country's multi-year plan that defines priorities, objectives, and approaches to strengthen immunization systems and contribute to toward achieving universal coverage. It aligns national goals with global and regional frameworks, such as IA2030, while adapting interventions to country-specific contexts. In the EMR, many countries are updating or developing their NIS, with explicit attention to vaccine prioritization, budgeting, and financing, particularly affordability, sustainability, and fiscal space. The NIS development is led by the EPI team with available support from 10.13039/100004423WHO and UNICEF at the country and regional levels. National Immunization Technical Advisory Groups (NITAGs), multidisciplinary expert bodies that provide independent, evidence-informed advice to the immunization programme, are also engaged [10]. NITAGs help translate global recommendations into national policies, considering local epidemiology, health system capacity, and financial constraints [10]. All EMR countries have functional NITAGs, though their functionality varies, and EPI teams serve as their secretariat [11].

Health economics is the application of economic studies that compare the costs and outcomes of at least two alternative programmes and provide quantitative evidence on the costs, benefits, and value-for-money of interventions such as vaccines [12]. A key concept of health economics is the opportunity cost, which recognizes that investing in one intervention (e.g., a new vaccine) may mean forgoing benefits from another [12]. Despite its relevance, health economics is underutilized in immunization programming and decision-making across the Middle East and North African countries [13]. Many of these countries lack institutional capacity or dedicated health economists to conduct or interpret economic evaluations [14]. A needs assessment in Jordan, Lebanon, the occupied Palestinian territories, and Türkiye highlights the urgent need for capacity development in this area [15].

Vaccine financing refers to the mobilization and allocation of financial resources -domestic, donor, or pooled- to procure, deliver, and sustain vaccines [16]. It is essential for programme sustainability and equitable access. IA2030 tracks progress through the indicator on domestic funding for vaccines, aligned with its Strategic Priority 6.3 [3]. Globally, vaccines account for just 0.3 % of current health expenditure and 0.02 % of GDP per capita, with significant variation by income group [17]. In the EMR, high-income countries (HICs) and upper middle-income countries (UMICs) finance over 90 % of vaccine expenditures, while low-income countries (LICs) cover only about 15 %, relying heavily on donor support [17]. This disparity highlights the financing vulnerabilities among lower-income EMR countries and the need for sustainable domestic resource mobilization.

This assessment aims to evaluate the use of health economics and vaccine financing evidence in national immunization programme and vaccine policy formulation across the EMR countries. It seeks to identify technical support needs and barriers and generate findings to guide tailored interventions that strengthen the use of economic evidence by the immunization programmes of ministries of health, and NITAGs for informed decision-making.

## 2 Methods

### 2.1 Study design

A cross-sectional online survey was conducted with a structured questionnaire, developed in Microsoft Forms and administered in English **(Annex 2)**. The survey focused on two domains. The first, health economics, covered cost–benefit analysis, cost-effectiveness analysis, and budget impact analysis, as these methods assess the value, efficiency, and economic implications of immunization policies. The second, vaccine financing, included financial forecasting and planning for immunization, financial sustainability, cost savings and procurement efficiencies, donor engagement, and the use of the NIS.Cost tool [18]. These aspects address the affordability, funding, and long-term viability of immunization programmes.

### 2.2 Participants

The EPI manager and NITAG chair in each country were invited to participate in the survey and were asked to jointly complete one questionnaire per country. They were purposively selected to provide responses on behalf of their EPI programme, rather than in an individual capacity.

### 2.3 Data collection

A web-based survey was distributed with the participants through email by the WHO Regional Office for the Eastern Mediterranean (EMRO). The survey remained open for completion in a three-week window (28 January to 18 February 2025) to ensure sufficient time for responses. It combined multiple-choice items, Likert scale (none, low, moderate, and high), and open-ended questions to capture both quantitative and qualitative insights.

The survey focused on five themes, each designed to capture critical aspects of health economics and vaccine financing capacity within national immunization programmes: (i) Current capacity in health economics and vaccine financing to assess institutional expertise and ability in conducting economic evaluations for immunization programmes; (ii) Training and capacity-building needs – To identify gaps and priority areas for training and workforce development; (iii) Technical support needs – To determine areas where countries require external technical assistance, such as study protocol development, data analysis, or evidence synthesis for decision-making; (iv) Data and evidence for decision-making – To evaluate the availability and use of economic evidence in vaccine policy decisions, including access to data sources; and (v) Strategic planning for vaccine financing – To explore the extent to which countries have developed strategic planning for financial sustainability of immunization programmes.

The final section provided an open-ended space for respondents to state their primary goals for incorporating health-economic and vaccine-financing considerations into immunization programmes over the next five years. The questionnaire content was informed by existing literature on immunization financing challenges and input from regional experts in health economics and vaccine policy [[19], [20], [21]].

### 2.4 Data analysis

Descriptive statistics (numbers and proportions) were summarized and presented. Findings from the Likert-scale questions were color coded in tables and figures to enhance clarity and visualization. To ensure confidentiality, countries are represented by codes and categorized by income levels according to the World Bank 2024–2025 categories: low-income (LIC), lower-middle-income (LMIC), upper-middle-income (UMIC), and high-income (HIC) [22].

The survey findings are accompanied by recommended action points at three levels - country, regional, and global, offering tailored support based on the specific needs identified at each level. This assessment was in line with best research practice, ensuring adherence to international research ethics norms [23,24].

## 3 Results

Sixteen out of 22 EMR countries responded to the survey (73 % response rate). Among the respondents, four were from LICs, six from LMICs, two from UMICs, and four from HICs. Non-responding countries included LIC (*n* = 1), LMICs (*n* = 3), and HICs (*n* = 2). Among the 16 completed surveys, seven responses (44 %) were jointly completed by the EPI manager and an NITAG chair, while six responses (37 %) were filled out solely by the EPI manager, two responses (13 %) were submitted by the NITAG secretariat (an EPI team member), and one response (6 %) was completed exclusively by an NITAG chair.

The rest of assessment findings are presented based on the five main themes of the survey.

### 3.1 Current capacity in health economics and vaccine financing

#### 3.1.1 Integration of health economics into immunization programmes and decision-making process

Three countries (19 %), one each from LIC, LMIC, and UMIC, reported full integration of health economics into immunization policy decision-making and programming. Four countries (25 %), one HIC, two LMICs, and one LIC, reported health economics being somewhat integrated. Five countries (31 %), two HICs, one UMIC, and two LMICs, indicated minimal integration, while the remaining four countries (25 %), one HIC, one UMIC, and two LICs, reported no integration at all.

In addition, only three countries (19 %), one each from HIC, UMIC, and LMIC, have a dedicated team or focal person for health economics in immunization programmes.

#### 3.1.2 Areas of health economics and vaccine financing utilized

The most utilized areas of health economics reported by the countries were vaccine costing (*n* = 10; 63 %), financial sustainability and resource mobilization (*n* = 7; 44 %), equity (*n* = 6; 38 %), burden of disease (n = 6; 38 %), and cost-effectiveness analysis (CEA) (*n* = 5; 31 %) (Table 1**)**.

**Table 1:**
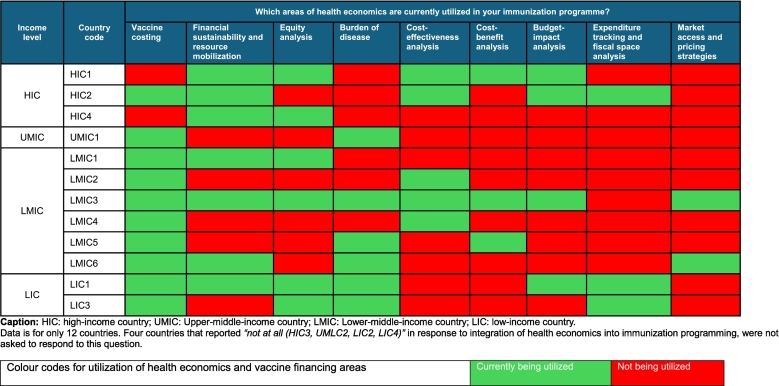
Health economics areas currently utilized by countries in the Eastern Mediterranean Region (EMR), February 2025 (*n* = 12).

### 3.2 Training and capacity building needs

#### 3.2.1 Current capacity in health economics

The health economics capacity across 16 countries varies significantly, ranging from none to high in different assessed areas. Among the four income groups, two HICs demonstrated strong capacity in utilizing health economics for immunization programmes, particularly in CEA, cost-benefit analysis, budget impact analysis, financial forecasting, and financial sustainability. Conversely, the other two HICs reported none to low capacity in all health economics and vaccine financing assessed areas **(**Table 2**)**.

**Table 2:**
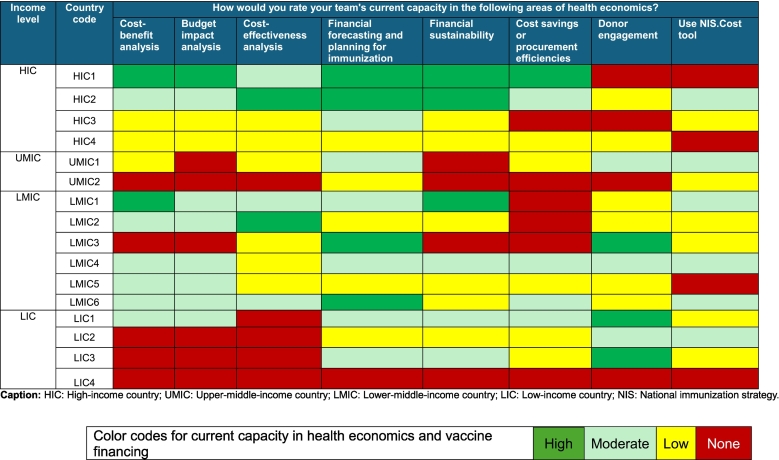
Current capacities of countries in health economics studies in Eastern Mediterranean Region (EMR), February 2025 (n = 16).

**Fig. 1:**
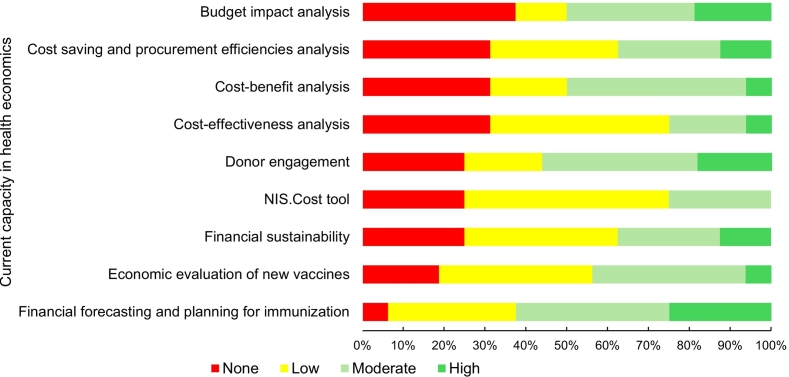
Variation in current health economics capacity by proportion of countries, EMR, February 2025 (*n* = 16).

UMICs generally reported none to moderate, while LMICs countries demonstrated a mix of none to high capacities across assessment areas. LICs reported none to low capacity in most health economics areas, except for financial forecasting for immunization and donor engagement, which were rated as moderate to high **(**Table 2**)**. Overall, ten countries (63 %) graded their financial forecasting and planning for immunization capacity as moderate or high. Both cost-benefit analysis and budget impact analysis were rated as moderate or high by eight (50 %) countries **(**Fig. 1**)**.

#### 3.2.2 Preference for capacity building format

Half of the countries (50 %) preferred a combination of in-person and online workshops, along with mentorship from experts. One country expressed a preference for an online format, while the remaining seven (44 %) countries were in favour of in-person capacity-building sessions. None preferred self-paced learning modules.

### 3.3 Technical support needs

**Fig. 2:**
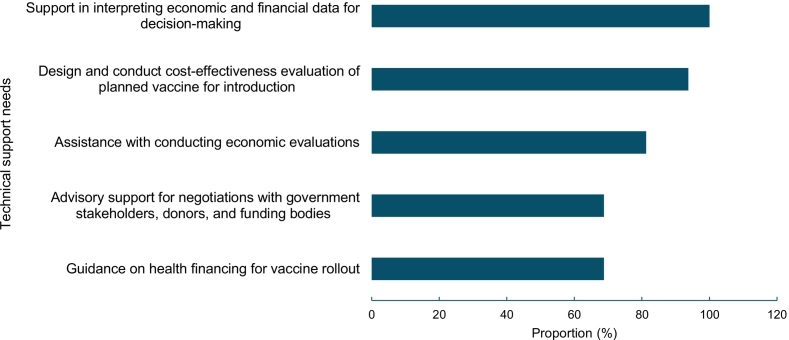
Technical and analytical support needs reported, Eastern Mediterranean Region (EMR), February 2025 (n = 16).

The top three identified areas for technical support, across all income levels, were: (1) support in interpreting economic and financial data for decision-making; (2) design and conduct cost-effectiveness evaluation of planned vaccine for introduction; and (3) assistance with conducting economic evaluations **(**Fig. 2**)**. Additional high-priority training and capacity building needs reported by certain countries were mathematical modeling, comparative analysis, and support with NIS development.

### 3.4 Data and evidence for decision-making

#### 3.4.1 Data source and accessibility

The main data sources that countries had access to and used to inform vaccine financing and health economic analysis include survey data (e.g., demographic and health survey and multiple indictors cluster survey reported (*n* = 13; 81 %), WHO and UNICEF data (*n* = 12; 75 %), and national health accounts (NHAs) (*n* = 11; 69 %). Seven countries (44 %) reported using data from other countries, whereas only four (25 %) utilized country-specific costing studies. Data for health economic evaluations were accessible to countries at variable extent. These were easily accessible in four countries (25 %), two HICs, one LMIC, one LIC; Somewhat accessible in eight countries (50 %), one HIC, one UMIC, five LIMC, and one LIC; Difficult to access in four countries (25 %), one HIC, one UMIC, and two LICs.

#### 3.4.2 Technical assistance on data generation and use

Three countries (19 %), one HIC and two LICs, rated their need as high in all identified areas, 1) collecting costing data, 2) improving quality of data, 3) utilization of data for programme planning, 4) collecting data for economic evaluation, 5) support with costing, financing, and budgeting of NIS, 6) vaccine product selection; and 7) vaccine switch costing, for technical assistance on data and evidence. Eight countries (50 %) from all income groups identified a high need for collecting data for vaccine economic evaluations **(**Table 3**).**

**Table 3:**
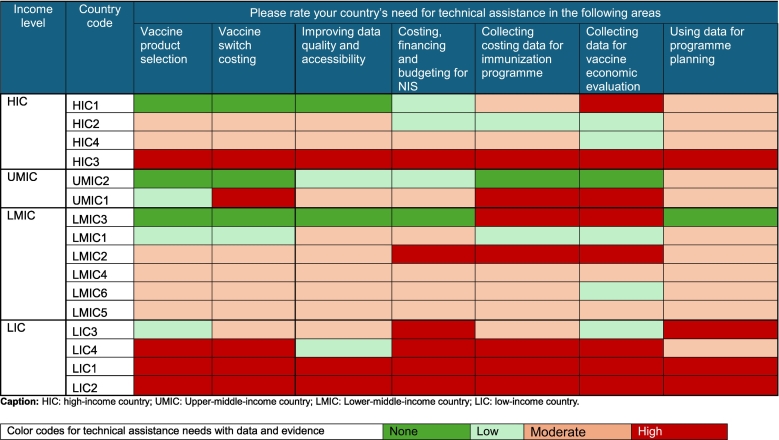
Eastern Mediterranean Region (EMR) countries' need for technical assistance with data and evidence decision-making (n = 16).

### 3.5 Strategic planning for vaccine financing

#### 3.5.1 Financing sources for vaccine procurement

Vaccine procurement is funded primarily through domestic government budgets in nine countries (56 %), four HICs, two UMICs, and three LMICs. Four countries (25 %), three LMICs and one LIC utilize a combination of domestic government funding and international donor support (e.g., Gavi, World Bank, WHO, UNICEF). Three LICs (19 %) remain dependent on international donors for vaccine procurement. Private sector contributions and out-of-pocket payments are non-existent in vaccine procurement in the region.

#### 3.5.2 Financing sources for immunization programme operation

Six countries (37 %), four HICs and two LMICs finance their immunization programme's operation entirely through domestic government funds. Another seven countries (44 %), two UMICs, three LMICs, and two LICs rely on a combination of domestic government funding and international donor support for programme operations. Meanwhile, three counties (19 %), two LICs and one LMIC are the only reported countries entirely dependent on international donors for the operation of their immunization programmes.

#### 3.5.3 Availability of strategic plan for vaccine financing

In response to questions about sustainable vaccine financing, countries demonstrated varying approaches to financing strategies; two HICs (12 %) have a fully implemented strategy, four countries (25 %) one UMIC, two LMICs, and one LIC are in the process of developing a strategy, while the remaining (63 %), two HICs, one UMIC, four LMICs, and three LICs, have no plans in place.

### 3.6 Primary goals in health economics and vaccine financing

HICs aim at creating sustainable systems for vaccine cost-effectiveness, investing in forecasting tools to prevent stock-outs or over-purchasing, and conducting health technology assessments before including vaccines in national immunization programmes. They focus on providing the best products at reasonable costs to protect nationals and residents from VPDs.

The UMICs prioritize the efficient use of available funds for vaccine procurement and operations, ensuring sustain funding for needed vaccines over the coming years, and securing funds for new vaccines. They also focus on developing NIS, advocating to decision-makers for costing the EPI, and introducing health economics into immunization programmes.

The LMICs emphasize sensitizing sub-national/provincial authorities about the Gavi exit strategy, introducing new vaccines as per NITAG recommendations, and sustaining vaccination coverage and EPI achievements. They aim at succeeding in transitioning to complete payment for vaccine and operational costs, maintaining efficiency in planning, and addressing vaccine pricing. Additionally, LMICs focus on improving healthcare access, ensuring sustainable funding for vaccination programmes, securing international support and partnerships, enhancing vaccine distribution, reducing dependency on external aids, and strengthening the national healthcare system.

Lastly, LICs prioritize increasing vaccination coverage for vulnerable populations and investing in health infrastructure to support long-term health security and resilience.

## 4 Discussion

The WHO EMRO needs assessment reveals significant disparities in integrating health economics into immunization programmes across the region. Only 19 % of countries reported full integration of health economics, highlighting systemic capacity gaps. The degree of integration does not correlate with income level, and aligns with global evidence that institutional support, political will, and stakeholder buy-in often play a more decisive role than income level in mainstreaming health economics into public health decision-making [25]. To address these disparities, targeted efforts are needed to build institutional capacity and design immunization programmes that explicitly incorporate equity considerations, ensuring that underserved populations, particularly those with higher disease burdens, benefit fully from vaccination efforts [26].

Only three countries reported having a dedicated health economics focal person or team, illustrating a broader regional gap, that is the lack of in-country expertise to lead, interpret, and apply economic evaluations. Only a third of countries reported using CEA as opposed to two-thirds using programming tools like vaccine costing and financing, and about half of the countries using vaccine forecasting tool. This suggests that the application of health economics in immunization programmes tends to focus more on meeting programmatic and operational needs rather than informing strategic decision-making. Costing and financial forecasting tools appear to be more commonly used, likely due to their relative accessibility and alignment with donor reporting requirements, such as those associated with Gavi funding applications.

Almost all respondents preferred in-person or blended learning over self-paced online modules, aligning with previous studies on decision-maker preference for training format [27]. It emphasizes the importance of interactive, mentored training approaches for building sustainable capacity. This preference is consistent with findings from earlier WHO and Gavi-supported regional training programmes, which have shown that peer learning and mentorship foster deeper engagement and better application of technical concepts [28]. Countries identified key areas for support in interpreting economic data, conducting CEAs, and preparing vaccine introduction dossiers. These functions are fundamental to evidence-informed policymaking but remain underdeveloped in much of the region. The results echo previous studies that identified persistent gaps between data availability and its actual use in health planning across LMICs [[29], [30], [31]].

Regarding financing vaccine supplies, LICs continue to rely heavily on donor funding, a dependency that threatens sustainability, especially as countries transition out of Gavi support. The absence of private sector or out-of-pocket contributions is a positive sign for equity but also signals the need for diversified and sustainable financing mechanisms. For operational immunization funding, LICs and some LMICs remain dependent on donor support. This vulnerability is further compounded by the fact that only two countries (both HICs) have fully developed vaccine financing strategies, while two-thirds reported having none. These findings are particularly concerning given the WHO Immunization Agenda 2030 (IA2030) emphasis on domestic financing as a cornerstone of resilient immunization systems [3].

All but one country reported a moderate to high need for technical assistance in using health economics data for programme planning and decision-making. This underscores the growing demand for integration and institutionalization of health economics into immunization programmes and highlights the need for coordinated efforts among stakeholders to foster a culture of evidence-informed decision-making in countries. Embedding the routine use of health economics in immunization programmes is essential for effective priority setting and programme implementation. In addition to strengthening internal capacity and infrastructure, more efforts should be made to improve access to, and the quality of, underlying cost, financing, and health outcomes data for economic evaluations [32]. Regardless of income group, countries reported varying levels of data availability and accessibility. More investigation and consultation will be needed to understand enablers and barriers to data use in individual countries.

Lastly, several countries reported none to low capacity in using the NIS.Cost tool, a tool developed by UNICEF, in collaboration with WHO and other partners, to support countries in estimating the costs of implementing their NIS for improved planning, budgeting, and resource mobilization. It is important to recognize that WHO and UNICEF have already been actively supporting this area through regional workshops and targeted country-level trainings as part of broader efforts to strengthen NIS development across the region.

This assessment has several limitations. Firstly, it relies on self-reported data, which may be subject to reporting and social desirability bias. Respondents may have over- or under-estimated their country's capacity and extent of integration based on perceived expectations or limited understanding. Secondly, the technical expertise of respondents varied considerably. Although the survey was intended to be jointly completed by NITAG chair and EPI manager, this occurred in only 44 % of cased. In 37 % of responses, the survey was completed solely by EPI manager, and 6 % solely by NITAG chair. This distribution may have biased the results toward programme operations and vaccine financing perspectives, with comparatively less emphasis on the health economics dimension of policy recommendations. In some instances, surveys may also have been completed by individuals with limited knowledge of health economics, potentially affecting the depth and accuracy of the responses. Thus, the results should be read cautiously. Thirdly, the assessment did not triangulate findings with independent data sources such as peer-reviewed publications, policy documents, or programme evaluations. Fourthly, the survey focused on national-level insights and did not assess subnational variations. In countries with decentralized health systems, significant disparities may exist at the provincial or district levels that this assessment does not capture. Finally, the assessment lacked a standardized definition of “integration,” with which we meant institutionalization of health economics in vaccine decision-making. We kept the definition open as *initialization* may be interpreted differently. However, this led to variation in responses as well. For instance, one country reported full integration of health economics into its immunization programme, but when asked about specific areas of application, responses ranged from “none” to “moderate”. This highlights the need for clearer conceptual framing in future assessments. Thus, the findings and conclusions should be interpreted with caution, particularly where inconsistencies exist.

Despite its limitations, this assessment offers several strengths. It is the first WHO Regional-specific initiative to systematically explore the integration of health economics and vaccine financing into immunization programmes, providing a regionally relevant baseline for planning and policy formulation. The assessment integrates both technical and strategic dimensions, including decision-making, financing, data use, and institutional capacity. This multidimensional approach ensures the findings are actionable and directly relevant to policymakers and immunization partners. In addition, the survey comprehensively addressed themes such as vaccine financing, technical expertise, training needs, and data application, providing a holistic understanding of the health economics landscape in immunization across the EMR countries. By gathering responses from national immunization programme teams and NITAG, the findings reflect on-the-ground realities at national level and not just external assumptions.

Finally, the results have already informed the design of the WHO EMRO roadmap for capacity strengthening in health economics and immunization programmes. Furthermore, the findings from this analysis can be relevant to shape global level priorities on country support, enhancing coordination and aligning global partners toward greater impact in countries. By aligning programmatic support with expressed needs, particularly for in-person, mentored learning, the assessment has laid the groundwork for sustainable, country-led progress toward more resilient and evidence-informed immunization systems. This assessment provides a foundation for future studies to explore issues in greater depth, such as reviewing published NITAG recommendations and examining the extent to which health economics is applied in those recommendations.

## 5 Conclusion and ways forward

The findings provided a comprehensive understanding of the current capacities, gaps, and areas for improvement in integrating health economics and vaccine finacing into immunization programmes across the EMR. Based on these insights, the following ways forward are proposed at the country, regional and global level.

***Countries:*** (i) Strengthen NITAG capacity by enhancing members' skill in interpreting and appraising economics evidence, preferably extend membership to include a health economist. (ii) Develop a skilled workforce in health economics within the ministries of health to support evidence-informed immunization decisions. (iii) Foster collaboration with academia to generate economic evidence for vaccines and immunization programmes. (iv) Promote financial sustainability through evidence-based policy dialogues and planning. (v) Enhance multi-sectoral engagement in immunization financing and policy development.

***Regional:*** (i) Continue providing technical support for 10.13039/501100003581NIS costing, budgeting and financing complemented by advocacy for resource mobilization for vaccine and immunizaton. (ii) Support evidence generation and use by assisting countries to conduct economic evaluations for immunization policy, ensure programme financial sustainability, and track immunization financing, including VPDs treatment costs and outbreak response expenditures. (iii) Enhance health economics capacity at the country level through training and dissemination of new economics evidence. (iv) Facilitate regional peer learning by establishing and maintaining a health economics network for country experts to share experiences, tools, and best practices.

***Global:*** (i) Provide normative, policy, and technical guidance to support countries in integrating health economics into immunization planning and decision-making processes. (ii) Coordinate global technical assistance efforts to strengthen in-country and regional capacity in health economics, ensuring alignment with gaps and priorities identified in the EMR. (iii) Promote alignment and coordination among global health partners and donors to support implementation of regional and national roadmaps for building health economics capacity in immunization. (vi) Enable cross-regional knowledge exchange through platforms such as communities of practice, joint trainings, and shared repositories of economic evidence, tools, and best practices in immunization economics and financing.

## CRediT authorship contribution statement

**Palwasha Anwari:** Writing – original draft, Visualization, Methodology, Investigation, Formal analysis, Conceptualization. **Gerald Sume:** Writing – review & editing, Conceptualization. **Wedyan Meshreky:** Writing – review & editing. **Nathalie Vande Maele:** Writing – review & editing. **So Yoon Sim:** Writing – review & editing. **Karene Hoi Ting Yeung:** Writing – review & editing. **Diana Kizza:** Writing – review & editing, Methodology. **Philipp Lambach:** Writing – review & editing. **Maarten Paul Maria Jansen:** Writing – review & editing. **Raymond Hutubessy:** Writing – review & editing. **Quamrul Hasan:** Writing – review & editing, Supervision, Conceptualization.

## Disclaimer

The authors are staff members and consultants of the World Health Organization. The authors alone are responsible for the views expressed in this article and they do not necessarily represent the decisions, policy or views of the World Health Organization.

## Declaration of competing interest

The authors declare that they have no known competing financial interests or personal relationships that could have appeared to influence the work reported in this paper.

## Data Availability

The data that has been used is confidential.

## Appendix A Supplementary data

Supplementary material Image 1

## References

[1] Shattock Andrew J., Johnson Helen C., Sim So Yoon (2024). Contribution of vaccination to improved survival and health: modelling 50 years of the expanded Programme on immunization. The Lancent.

[2] Sim So Yoon, Watts Elizabeth, Constenla Dagna, Brenzel Logan, Patenaude Bryan N. (2020). R*eturn on investment from immunization against 10 pathogens in 94 low- and middle-income countries, 2011–30*. Health Aff.

[3] WHO (2020). https://www.who.int/docs/default-source/immunization/strategy/ia2030/ia2030-document-en.pdf.

[4] EMRO (2025).

[5] United Nations. Department of economic and social affairs population division. World population prospects (2022). https://population.un.org/wpp/downloads?folder=Documentation&group=Documentation.

[6] Anna Minta A., Antoni Sebasgtien, Matt Ferrari M. (2024). Progress toward measles Eliminiation-worldwide. MMWR Morb Mortal Wkly Rep.

[7] Ozawa S., Clark S., Portnoy A., Grewal S., Stack M.L., Sinha A. (2017). Estimated economic impact of vaccinations in 73 low- an middle-income countries 2001–2020. Bull World Health Organ.

[8] Bencina G., Ugrekhelidze D., Shoel H., Oliver E., Meiwald A., Hughes R. (2024). The indirect costs of vaccine-preventable cancer mortality in the Middle East and North Africa (MENA). J Med Econ.

[9] WHO (2025). https://iris.who.int/handle/10665/357686.

[10] Henaff L., Zavodska D., Melgar M., Fihman J. (2025). The role of NITAGs in government decisions on vaccine use: insights from the Fifth Global NITAG Network meeting. Lancet Infect Dis.

[11] Sume E.G., Hasan Q., Shefer A., Henaff L. (2023). Region-wide assessment of National Immunization Technical Advisory Groups (NITAGs) using the NITAG maturity assessment tool (NMAT)– experience from the eastern Mediterranean region of the World Health Organization. Vaccine.

[12] WHO (2019).

[13] Nagi M.A., Luangsinsiri C., Thavorncharoensap M. (2021). A systematic review of economic evaluations of vaccines in Middle East and North Africa countries: is existing evidence good enough to support policy decision-making?. Expert Rev Pharmacoecon Outcomes Res.

[14] Nagi M.A., Luangsinsiri C., Thavorncharoensap M. (2021). A scoping review of health economic evaluation in the World Health Organization eastern Mediterranean region. Expert Rev Pharmacoecon Outcomes Res.

[15] Gheorghe A., Gad M., Ismail S.A., Chalkidou K. (2020). Capacity for health economics research and practice in Jordan, Lebanon, the occupied Palestinian territories and Turkey: needs assessment and options for development. Health Res Policy Syst.

[16] WHO (2022).

[17] WHO (2025). WHO/UNICEF Joint Reporting Process [Internet]. https://www.who.int/teams/immunization-vaccines-and-biologicals/immunization-analysis-and-insights/global-monitoring/who-unice.

[18] UNICEF (2022). NIS.Cost app and user guide from UNICEF. https://immunizationeconomics.org/recent-activity/2022/4/1/niscost-app-and-user-guide/.

[19] McLaughlin J.M., McGinnis J.J., Tan L. (2015). Estimated human and economic burden of four major adult vaccine-preventable diseases in the United States, 2013. J Prim Prev.

[20] Cernuschi T., Gaglione S., Bozzani F. (2018). Challenges to sustainable immunization systems in Gavi transitioning countries. Vaccine.

[21] Zhang X., Chen S., Zhu K., Tang S. (2025). Financing the introduction of new vaccines to the national immunisation programme in China: challenges and options for action. BMJ Glob Health.

[22] World Bank (2025). World Bank Country Classification by income level for 2024–2025. https://blogs.worldbank.org/en/opendata/world-bank-country-classifications-by-income-level-for-2024-2025.

[23] UN. (2022). World population prospects 2022. CVS format.

[24] WHO (2023). https://www.who.int/about/ethics.

[25] Bouckley T., Peiris D., Nambiar D. (2025). Addressing health equity during design and implementation of health system reform initiatives: a scoping review and framework. Int J Equity Health.

[26] Patikorn C., Cho J.Y., Lambach P., Hutubessy R., Chaiyakunapruk N. (2023). Equity-informative economic evaluations of vaccines: a systematic literature review. Vaccines (Basel).

[27] Leask Julie, Christou-Ergos Maria, Abdi Ikram, Mboussou Franck, Sabahelzain Majdi M., Wiley Kerrie E. (2025). Informing the development of transmission modelling guidance for global immunization decision-making: a qualitative needs assessment. Vaccine.

[28] Gavi (2023). Learning and performance management (LPM): improving health worker performance and health outcomes. https://www.gavi.org/programmes-impact/types-support/health-system-and-immunisation-strengthening/lpm.

[29] Sharkawy M.N., Dastan I. (2021). A scoping review of health economic evaluation in the World Health Organization eastern Mediterranean region. Expert Rev Pharmacoecon Outcomes Res.

[30] Byrne E., Heywood A. (2023). Use of routine health information systems data in developing and monitoring district and facility health plans: a scoping review. BMC Health Serv Res.

[31] Kumar M., Gotz D., Nutley T., Smith J.B. (2018). Research gaps in routine health information system design barriers to data quality and use in low- and middle-income countries: a literature review. nt J Health Plann Mgmt.

[32] Rafferty E., Reifferscheid L., Assi A., MacDonald S.E. (2022). Using health economics to inform immunization policy across all levels of government. Pharmacoecon Open.

