# Supplementary material for: Health economics and vaccine financing in the eastern Mediterranean region: A needs assessment
Source: Vaccine. 2025 Oct 24;65:None. doi: 10.1016/j.vaccine.2025.127780 (PMC12550511; doi:10.1016/j.vaccine.2025.127780)
Supplement: Supplementary file 1 — Supplementary material [file mmc1.docx]

**Health economics and vaccine financing in the eastern Mediterranean region: A needs assessment**

**Appendix A supplement data**

**Annex 1. Countries and territories of the World Health Organization (WHO) Eastern Mediterranean Region (EMR)**

Annex 1 presents the list of countries comprising the WHO Eastern Mediterranean Region (EMR), arranged in alphabetical order.

1. Afghanistan
2. Bahrain
3. Djibouti
4. Egypt
5. Iran (Islamic Republic of)
6. Iraq
7. Jordan
8. Saudi Arabia
9. Kuwait
10. Lebanon
11. Libya
12. Morocco
13. occupied Palestinian territory
14. Oman
15. Pakistan
16. Qatar
17. Somalia
18. Sudan
19. Syrian Arab Republic
20. Tunisia
21. United Arab Emirates
22. Yemen

## **Annex 2**

Annex 2 provides the needs assessment survey questionnaire employed to collect data for this study.

## survey questionnaire

Developed by: IVP/DCD, WHO EMRO

Final version: January 20, 2025

**Title: Health economics and vaccine financing needs assessment survey, Eastern Mediterranean Region (EMR), 2025**

**Introduction**

This survey is designed to assess the health economics and vaccine financing needs of countries within the Eastern Mediterranean Region (EMR) of the World Health Organization (WHO). The findings will provide valuable insights into existing challenges, gaps, and opportunities to strengthen health systems, particularly in immunization programmes. By identifying country specific priority areas, this assessment aims to provide tailored support to countries to address identified health economics related gaps. Your input is vital to shape evidence-based policy and tailored programmatic support.

Just a single questionnaire should be filled per country. This will take about 15 minutes.

Thank you for your participation in this survey!

**General Information**

1. Country name (select from dropdown button)
2. Afghanistan
3. Bahrein
4. Djibouti
5. Egypt
6. Iran, Islamic Republic of
7. Iraq
8. Jordan
9. Kingdom of Saudia Arabia (KSA)
10. Kuwait
11. Lebanon
12. Libya
13. Morocco
14. Occupied Palestinian Territory
15. Oman
16. Pakistan
17. Qatar
18. Somalia
19. Sudan
20. Syria
21. Tunisia
22. United Emirates Arab (UEA)
23. Yemen
24. Respondent functional title (select all that that apply) - Checkbox
    - National EPI Manager
    - NITAG Chair/Member
    - National EPI Manager and NITAG Chair/Member
    - Others (specify):__________________________________________

**Section 1: Current capacity in health economics and vaccine financing**

1. To what extent is health economics integrated into your country’s immunization programme?
   - Not at all
   - Minimal integration
   - Somewhat integrated
   - Fully integrated

**Note**: If your response is “Not at all” move to question 5.

1. Which areas of health economics are currently utilized in your immunization programme? (Select all that apply) – **[checkbox]**
   - Vaccine costing
   - Cost-effectiveness analysis
   - Cost-benefit analysis
   - Budget impact analysis
   - Financial sustainability and resource mobilization
   - Equity analysis
   - Expenditure tracking and fiscal space analysis
   - Market access and pricing strategies
   - Burden of disease assessment
   - Other (please specify): ____________________
2. Does your national immunization programme have a dedicated team or focal person for health economics related issues/topics?
   - Yes
   - No
3. What are the primary sources of funding for vaccine procurement in your immunization programme? (Select all that apply) – **[checkbox]**
   - Domestic government budget
   - International donors (e.g., Gavi, World Bank, WHO, UNICEF, etc.)
   - Private sector contributions
   - Out-of-pocket payments by citizens
   - Other (please specify): _____________________
4. What are the primary sources of funding for your immunization programme’s operation? (Select all that apply) – **[checkbox]**
   - Domestic government budget
   - International donors (e.g., Gavi, World Bank, WHO, UNICEF, etc.)
   - Private sector contributions
   - Out-of-pocket payments by citizens
   - Other (please specify): _____________________

**Section 2: Training and capacity building needs**

1. How would you rate your team's current capacity in the following areas of health economics?

| **Health economics areas to assess country capacity building needs** | **None** | **Low** | **Moderate** | **High** |
| --- | --- | --- | --- | --- |
| Cost-effectiveness analysis |  |  |  |  |
| Cost-benefit analysis |  |  |  |  |
| Budget impact analysis |  |  |  |  |
| Economic evaluation of new vaccines |  |  |  |  |
| Financial forecasting and planning for immunization |  |  |  |  |
| Financial sustainability |  |  |  |  |
| Donor engagement |  |  |  |  |
| Data analysis for health economic studies |  |  |  |  |
| How to use NIS.Cost to cost the National Immunization Strategy (NIS), NIS strategic plan |  |  |  |  |
| Cost savings or procurement efficiencies |  |  |  |  |

1. Indicate any other specific health economics related capacity building needs for your national immunization programme or NITAG not listed above?

________________________________________________________________________________________________________________________________________________________________

1. Preferred format for training (Select all that apply): **[checkbox**]
   - In-person training workshops
   - Online courses
   - Mentorship from health economics experts
   - Self-paced learning modules
   - Combination of the above

**Section 3: Technical and analytical support needs**

1. Which types of technical support would be most beneficial to your immunization programme? (Select all that apply) **[checkbox**]
   - Assistance with conducting economic evaluations
   - Support in interpreting economic and financial data for decision-making
   - Design and conduct cost-effectiveness evaluation of planned vaccine for introduction
   - Guidance on health financing for vaccine rollout
   - Advisory support for negotiations with government stakeholders, donors, and funding bodies
2. Indicate any other specific health economics related technical and analytical support needs for your national immunization programme or NITAG not listed above?

________________________________________________________________________________________________________________________________________________________________

**Section 4: Data and evidence for decision-making**

1. What sources of data are currently used to inform vaccine financing and health economic analyses? (Select all that apply) **[checkbox]**
   - National Health Accounts
   - WHO and UNICEF data
   - Survey data (e.g., DHS, MICS)
   - Costing studies specific to our country
   - Data from other countries in the region
   - Other (please specify): __________________________
2. How accessible is data for performing economic evaluations in your country?
   - Easily accessible
   - Somewhat accessible
   - Difficult to access
   - Not accessible at all
3. Please rate your country’s need for technical assistance in the following areas:

| **Country’s need for technical assistance on data and evidence decision making and costing** | **None** | **Low** | **Moderate** | **High** |
| --- | --- | --- | --- | --- |
| Collecting and analyzing costing data for immunization programme |  |  |  |  |
| Improving data quality and accessibility |  |  |  |  |
| Using data for programme planning and decision-making |  |  |  |  |
| Collecting and analyzing data for vaccine economic evaluation |  |  |  |  |
| Costing, financing and budgeting for NIS |  |  |  |  |
| Vaccine product selection |  |  |  |  |
| Vaccine switch costing |  |  |  |  |

**Section 5: Strategic planning for vaccine financing**

1. Has your country developed a sustainable vaccine financing strategy?
   - Yes, developed and fully implemented
   - Yes, developed and implementation ongoing
   - No, but currently under development
   - No, no plans at present

**Country specific additional input**

1. What are your country’s primary goals in health economics and vaccine financing over the next 5 years? (Open text response)

________________________________________________________________________________________________________________________________________________________________

1. Are there any additional needs, challenges, or comments you would like to share regarding health economics and vaccine financing? (Open text response)

________________________________________________________________________________________________________________________________________________________________

**End of the survey.**
